# Double-Barrel Uro-Colostomy Versus Ileal Conduit for Urinary Diversion After Pelvic Exenteration: A Systematic Review and Meta-Analysis of Comparative Outcomes

**DOI:** 10.3390/cancers17213479

**Published:** 2025-10-29

**Authors:** Ahmed Salama, Gavin Calpin, Mahmoud Salama, Ben Creavin, Patrick J. Maguire, Peter Lonergan, Jonathan Cho, Feras Abu Saadeh, Louise McLoughlin, Tarik Sammour, Michael E. Kelly

**Affiliations:** 1 Royal College of Surgeons Ireland, 123 St. Stephens Green, D02 YN77 Dublin, Ireland; 2 Department of Colorectal Surgery, St. James’ Hospital, D08 NHY1 Dublin, Ireland; 3 Department of Surgery, School of Medicine, Trinity College Dublin, D02 PN40 Dublin, Ireland; 4 Department of Gynecology, St. James’ Hospital, D08 NHY1 Dublin, Ireland; 5 Trinity St. James Cancer Institute, St. James’ Hospital, D08 NHY1 Dublin, Ireland; 6 Urology Unit, Department of Surgery, Royal Adelaide Hospital, Adelaide, SA 5000, Australia; 7 Colorectal Unit, Department of Surgery, Royal Adelaide Hospital, Adelaide, SA 5000, Australia; 8 Adelaide Medical School, Faculty of Health and Medical Sciences, University of Adelaide, Adelaide, SA 5005, Australia

**Keywords:** Pelvic Exenteration, urinary reconstruction, ileal conduit, double-barrelled uro-colostomy, complications

## Abstract

**Simple Summary:**

Pelvic exenteration is a major operation used to treat advanced cancers in the pelvis. After this surgery, patients need new pathways for both urine and stool to leave the body. The traditional method uses two separate stomas—one for urine and one for stool—while a newer method, called double-barrel uro-colostomy, combines both into a single stoma. This study reviewed and combined results from previous research comparing these two techniques. We found that the single-stoma approach had fewer urine leaks and similar overall complication rates. This suggests it may be a safe and simpler alternative for selected patients. These findings may help guide surgeons in choosing the most suitable reconstruction method and encourage further research into long-term outcomes and quality of life.

**Abstract:**

Introduction: Pelvic exenteration is a radical operation for advanced or recurrent pelvic malignancies, requiring urinary and faecal diversion. The ileal conduit (IC) remains the standard urinary diversion, while the double-barrel uro-colostomy (DBUC) has re-emerged as an alternative that avoids small bowel anastomosis and consolidates diversion into a single stoma. Aims: To evaluate comparative outcomes of DBUC versus IC to clarify relative risks and potential benefits. Methods: A systematic review and meta-analysis was conducted in accordance with PRISMA guidelines and registered on PROSPERO (CRD420251090885). PubMed, Scopus, EMBASE, and Medline were searched to March 2025 for studies directly comparing DBUC and IC following pelvic exenteration. Eligible studies reported perioperative or urological outcomes. Results: Four retrospective studies (164 patients; DBUC 88, IC 73) were included. Urinary leak was lower with DBUC (10.2% vs. 15.1%), with pooled analysis showing a higher risk in IC (RR 2.52, 95% CI 1.02–6.20, *p* = 0.04). Pyelonephritis (42.0% vs. 15.3%; RR 1.37, *p* = 0.24) and electrolyte derangements (20.6% vs. 15.6%; RR 1.21, *p* = 0.64) did not differ significantly. Rates of urinary and enteric fistulas were similar. Clavien–Dindo grade III (42.1% vs. 37.1%) and grade IV complications (17.1% vs. 24.2%) were also comparable between groups. Conclusion: DBUC is a feasible alternative to IC after pelvic exenteration, with reduced urinary leak rates and comparable morbidity. Its single-stoma approach may offer patient-centred advantages. Larger prospective studies incorporating long-term and quality-of-life outcomes are needed.

## 1. Introduction

Pelvic exenteration is a highly morbid surgical procedure typically reserved for patients with locally advanced or recurrent malignancies of the pelvis—most commonly of colorectal, gynaecological, or urological origin [1]. It involves *en bloc* resection of involved pelvic organs and may be performed with curative intent or for palliation in carefully selected patients with symptomatic disease. The complexity of exenteration extends beyond the resection itself, as it frequently necessitates both urinary and faecal diversion. While morbidity rates for this high-risk procedure are commonly reported to approach 50% in contemporary series [2,3], the PelvEx Collaborative group described a wider range of 32% to 84% in an international multicentre cohort study [4,5]. The ileal conduit (IC) reconstruction has long been the standard method of urinary reconstruction, favoured due to widespread surgeon familiarity and a well-established safety profile [6,7]. However, an alternative technique known as the double-barrel uro-colostomy (DBUC), which combines urinary and faecal diversion into a single stoma, has garnered renewed interest in recent years due to its potential to simplify reconstruction with one less anastomosis, preserve one side of the abdominal wall musculature, and potentially enhance postoperative recovery [8].

The concept of a “wet colostomy” dates back to 1948, when Brunschwig first described total pelvic exenteration (TPE) as a treatment for advanced pelvic malignancies [9]. His original technique involved a single-stoma reconstruction using bilateral refluxing uretero-colostomies anastomosed proximally to an end colostomy [10]. Although this approach simplified diversion, it was soon associated with serious complications, including ascending pyelonephritis, electrolyte imbalances, and frequent malodorous watery diarrhoea. Mortality directly related to urinary diversion in Brunschwig’s early series approached 9% [11], and some reports quoted overall mortality for TPE up to 30%, driven by both surgical complexity and aggressive disease [12,13]. Due to these high complication rates, the technique was largely abandoned in favour of dual-stoma reconstructions—most commonly the ileal conduit (IC) for urinary diversion combined with a separate colostomy [14]. However, even with IC, urinary tract infections, including pyelonephritis, remain a concern, with rates reported as high as 23% [15,16].

In 1989, Carter and colleagues introduced the double-barrel wet colostomy (DBWC) as a modernised alternative to the traditional wet colostomy and the dual-stoma model [17,18]. This refined technique maintains a single abdominal stoma but anatomically separates the urinary and faecal streams via a proximal urinary conduit and a distal faecal limb of a divided colon. Unlike the original wet colostomy, DBWC avoids continuous diarrhoea, instead offering patients continuous urinary drainage and intermittent, more typical semi-formed bowel movements. Importantly, the DBWC approach also eliminates the need for a small bowel anastomosis, which can be particularly beneficial in patients who are malnourished, irradiated, or undergoing prolonged and technically demanding operations [19]. These theoretical and practical advantages have led to renewed interest in DBWC as a streamlined and potentially less morbid reconstruction in the setting of pelvic exenteration [20]. To clarify that the stoma is not “wet” in the traditional sense, and for accuracy of anatomical description, naming was changed to double-barrel uro-colostomy (DBUC) in some contemporary series [21].

Despite promising data, DBUC reconstruction remains underutilized, largely due to persistent anecdotal concerns over infectious and metabolic complications, and a lack of large-scale comparative data. Despite isolated reports and narrative reviews describing the double-barrel uro-colostomy, there has been no prior quantitative synthesis directly comparing its outcomes with those of the ileal conduit following pelvic exenteration. This systematic review and meta-analysis uniquely consolidates the existing comparative evidence to clarify relative risks and potential advantages of each technique, aiming to guide reconstructive decision-making in this complex surgical setting.

## 2. Methods

This systematic review was performed in accordance with the Preferred Reporting Items for Systematic Reviews and Meta-Analyses (PRISMA) guidelines [22]. The study was registered on PROSPERO (registration number: CRD420251090885) Each author contributed to formulating the study protocol. Local institutional ethical review and approval was not required.

### 2.1. PICO

Using the PICO framework, the aspects the authors wished to address were:

Population—Patients undergoing urinary reconstruction following pelvic exenteration for advanced or recurrent pelvic malignancies.

Intervention—Double-barrel uro-colostomy (DBUC) as a method of urinary and faecal diversion.

Comparison—Ileal conduit (IC) with a separate colostomy as the standard method of diversion.

Outcomes—Length of hospital stay, operative time, urological complications such as urinary leaks, pyelonephritis, and electrolyte disturbances, and bowel-related complications.

### 2.2. Search Strategy

An electronic search of PubMed, Scopus, EMBASE, and Medline was carried out to identify eligible studies. Two independent reviewers (AS, MS) conducted the search using a predefined strategy developed by a senior author. Any discrepancies were resolved through consensus discussion, with arbitration by a senior author (MK) when required. The search terms and Boolean operators included: (“pelvic exenteration” OR “pelvic exenterations”) AND (“urinary diversion” OR “urinary reconstruction”) AND (“double-barrel wet colostomy” OR “double barrel wet colostomy” OR “DBUC” OR “uro-colostomy”) AND (“ileal conduit” OR “Bricker conduit”). Only English-language publications were considered, and no restriction was applied to the year of publication. Duplicate records were removed manually before title screening. Abstracts and full texts of potentially relevant studies were then reviewed in detail. The final database search was completed on 5 March 2025.

### 2.3. Inclusion and Exclusion Criteria

Studies were included if they reported on patients who underwent pelvic exenteration for advanced or recurrent pelvic malignancy and received urinary reconstruction using either the double-barrel uro-colostomy (DBUC) or ileal conduit (IC) technique, with direct comparative data available between the two groups. Eligible study designs included original research articles (prospective or retrospective cohort studies) published in the English language that reported at least one perioperative, urological, or overall morbidity outcome of interest.

Exclusion criteria comprised case reports, review articles, technical descriptions without outcome data, and conference abstracts lacking full-text information. Studies were also excluded if urinary diversion was performed for non-exenteration indications, or if data could not be stratified by reconstruction type.

### 2.4. Data Extraction and Quality Assessment

Each reviewer independently assessed the retrieved manuscripts to ensure all inclusion criteria were met before extracting the following data: first author, year of publication, study design, country or institution, and study title. Clinical data included the number of patients in each group (DBUC vs. IC), indication for pelvic exenteration (e.g., cancer type or recurrence), type of exenteration (anterior, posterior, or total), and prior radiotherapy status. Surgical details such as the urinary diversion technique, operative time, and length of hospital stay were recorded. Outcome measures included urological complications (e.g., urinary leak, pyelonephritis, ureteric stricture), overall complication rates classified by Clavien–Dindo, postoperative mortality, and duration of follow-up. Oncological outcomes, such as recurrence or survival, were also noted if reported. Risk of bias and methodology quality assessment was performed in concordance with the Newcastle-Ottawa scale [23] (Supplementary Figures S1 and S2). In case of discrepancies in opinion between the reviewers, a third reviewer was asked to arbitrate.

### 2.5. Statistical Analysis

Outcomes were analysed as dichotomous variables and reported as risk ratios (RR) with 95% confidence intervals (CIs), calculated using the Mantel–Haenszel method. Statistical heterogeneity was assessed using the I^2^ statistic. In accordance with the Cochrane Handbook, values above 50% were considered to represent substantial heterogeneity, prompting the use of a random-effects model; otherwise, a fixed-effects model was applied. All significance tests were two-sided, with *p*-values < 0.05 considered statistically meaningful. Risk ratios were calculated in the direction of IC versus DBUC. All meta-analyses were conducted using Review Manager (RevMan) Version 5.4 (Nordic Cochrane Centre, Copenhagen, Denmark).

## 3. Results

### 3.1. Literature Search

**Figure 1:**
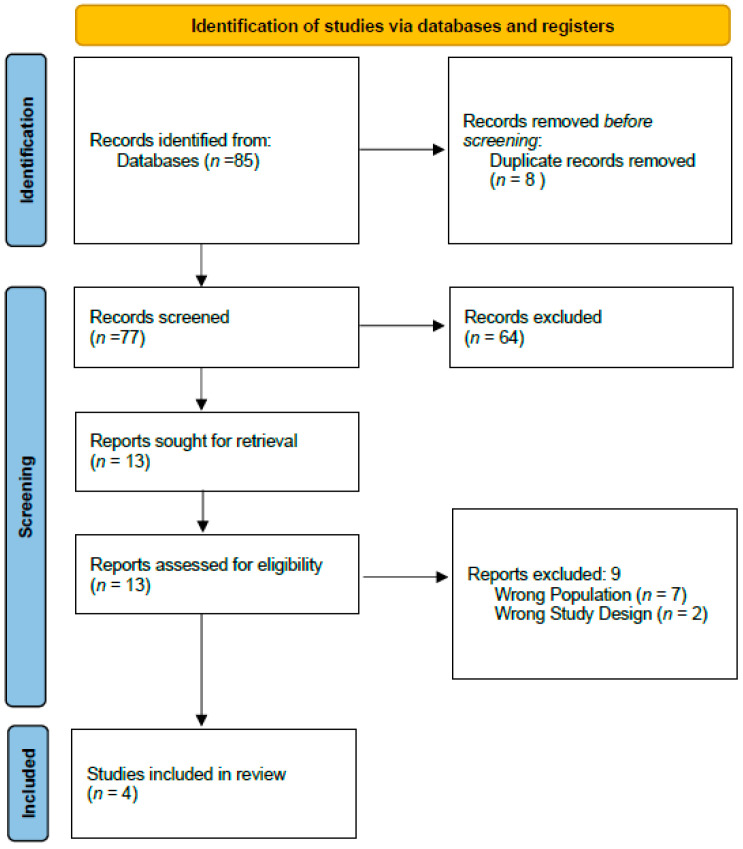
PRISMA flow diagram of study identification.

Overall, 85 studies were identified and 8 duplicates were removed. After screening titles, 64 studies were excluded. Of the remaining 13 studies, 9 were eliminated after the abstracts were reviewed. Four full texts were reviewed and were all included. The study identification is summarised in the PRISMA flow diagram (Figure 1).

### 3.2. Study Characteristics

Four retrospective cohort studies were included in this review: Lago et al. [24], Chokshi et al. [25], Backes et al. [20], and Nguyen et al. [19] (Supplementary Table S1). A total of 164 patients were included in this review, with 73 (44.51%) undergoing IC and 91 (55.49%) receiving DBUC. The most common tumour origin was colorectal in 60 patients (36.6%), followed by gynaecological malignancies in 91 patients (55.5%), urological in 6 patients (3.7%), anal squamous cell carcinoma in 2 patients (1.2%), benign in 1 patient (0.6%), and suspected malignancy in 1 patient (0.6%).

### 3.3. Baseline Characteristics

Across the four included studies, baseline demographics and operative features showed important differences between double-barrel uro-colostomy (DBUC) and ileal conduit (IC) patients as summarized in Table 1. DBUC cohorts were generally younger, with reported mean ages ranging from 56–62 years compared to 57–67 years for IC. BMI was inconsistently reported but broadly similar [25,26,27,28,29]. Preoperative radiotherapy was more frequent in DBUC, reaching 77–94% versus 60–65% for IC, though Backes et al. reported 100% in both groups. DBUC patients more often underwent total pelvic exenteration, while IC cohorts included a greater mix of anterior or modified procedures.

There was significant variation in reported operative times between both groups. Nguyen et al. reported a mean operative time of 566 min for DBUC compared to 415 min for IC, whereas Backes et al. paradoxically reported longer operative times in the IC group (720 min vs. 610 min for DBUC). Chokshi et al. found comparable operative durations between the two groups (approximately 547–549 min).

Length of stay (LOS) showed inconsistent trends. In Nguyen et al., LOS was slightly longer in the DBUC group (18.5 vs. 16 days), while in Backes et al., IC patients had longer stays (26 vs. 14.5 days). Lago et al. also reported longer LOS in the IC group (22 vs. 16 days), and Chokshi et al. found median LOS to be similar across groups (14–16.5 days).

Flap reconstruction was more commonly employed in patients undergoing DBUC. In Nguyen et al., 93.7% of DBUC patients required flap reconstruction compared to only 45.5% in the IC group. Lago et al. reported various flap types, including e-VRAM (30%), omental (44%), V-Y (7%), and gracilis flaps (3%), although this was not stratified by reconstruction type. The IC groups in other studies had either low or unreported flap usage.

### 3.4. Perioperative Morbidity

**Figure 2:**
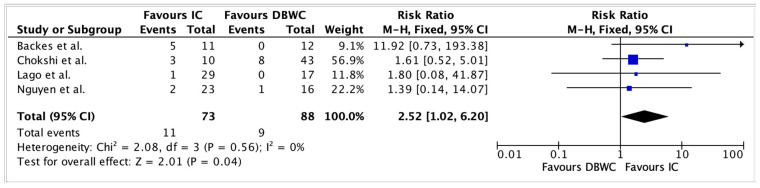
Urinary leak was significantly reduced in the DBUC group compared to the IC group (10.23% vs. 15.07%, RR 2.52, 95% CI 1.02–6.20, *p* = 0.04) [19,20,24,25].

Table 2 summarises urological and enteric complications across the included studies. Urinary leak rates were 10.23% (n = 9/88) in the DBUC group, compared to 15.07% (n = 11/73) in the IC group. Pooled analysis of urinary leaks demonstrates a significantly higher risk in the IC group compared to DBUC, with a risk ratio of 2.52 (95% CI: 1.02–6.20, *p* = 0.04) as shown in Figure 2. There was no significant heterogeneity among studies (I^2^ = 0%), supporting the consistency of this finding.

**Figure 3:**
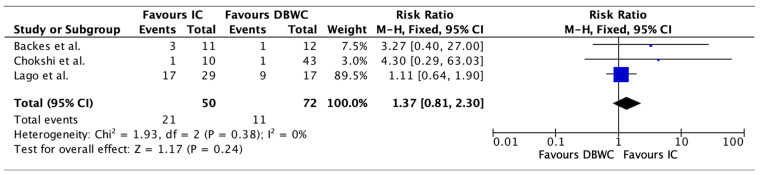
Pyelonephritis rates were higher in the IC group compared to the DBUC group (42.00% vs. 15.28%, RR 1.37, 95% CI: 0.81–2.30, *p* = 0.24) [20,24,25].

Pyelonephritis rates were variably reported across the studies. Pyelonephritis rates were 42.00% (n = 21/50) in the IC group compared to 15.28% (n = 11/72) in the DBUC group. Pooled analysis of pyelonephritis rates demonstrates no significant difference in the IC group compared to DBUC, with a risk ratio of 1.37 (95% CI: 0.81–2.30, *p* = 0.24) as shown in Figure 3. There was no significant heterogeneity among studies (I^2^ = 0%), supporting the consistency of this finding.

**Figure 4:**
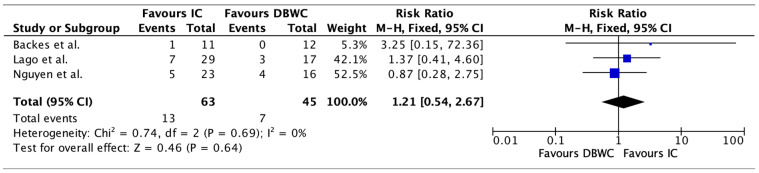
Electrolyte Derangement rates were similar in the IC and DBUC groups (20.63% vs. 15.56%, RR 1.21, 95% CI 0.54–2.67, *p* = 0.64) [19,20,24].

Electrolyte derangement rates were similar in the IC (20.63%, n = 13/63) and DBUC groups (15.56%, n = 7/45). Pooled analysis of electrolyte derangement rates demonstrates a risk ratio of 1.21 (95% CI 0.54–2.67, *p* = 0.64) as demonstrated in Figure 4. There was no significant heterogeneity among studies (I^2^ = 0%), supporting the consistency of this finding.

The incidence of urinary fistulas was 13.33% (n = 8/60) in the DBUC group and 15.38% (n = 6/39) in the IC group. Similarly, the incidence of enteric fistulas was 19.74% (n = 15/76) in the DBUC group, and 16.13% (n = 10/62) in the IC group.

### 3.5. Clavien–Dindo Postoperative Complications

**Figure 5:**
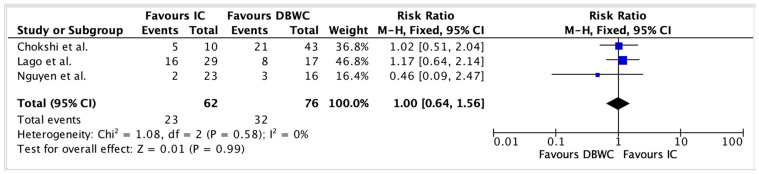
No statistically significant difference in Grade III complication rates between DBUC and IC (RR = 1.00, 95% CI 0.64–1.56, *p* = 0.99) [19,24,25].

Postoperative morbidity was assessed using the Clavien–Dindo classification (Supplementary Table S2). Grade III complications were observed in 32 of 76 (42.1%) DBUC patients and 23 of 62 (37.1%) IC patients. Pooled results from Figure 5 indicated a similar risk of Grade III complications in both groups, with a RR of 1.00 (95% CI: 0.64–1.56, *p* = 0.99).

**Figure 6:**
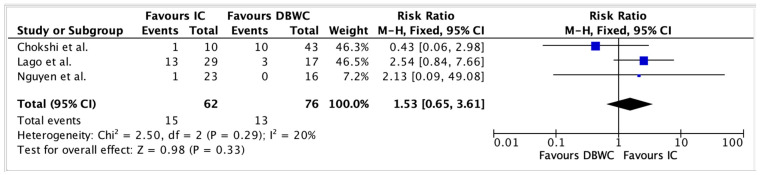
No statistically significant difference in Grade IV complication rates between DBUC and IC (RR = 1.53, 95% CI 0.68–3.43, *p* = 0.33) [19,24,25].

Grade IV complications occurred in 13 of 76 (17.1%) DBUC patients and 15 of 62 (24.2%) IC patients. As seen in Figure 6, pooled analysis showed a non-significant trend favouring DBUC, with a RR of 1.53 (95% CI: 0.65–3.61, *p* = 0.33).

## 4. Discussion

Pelvic exenteration represents the most radical but potentially curative surgical option for locally advanced or recurrent pelvic malignancies, with urinary diversion constituting one of the most critical and complication-prone aspects of reconstruction [1,2,5,6]. This systematic review and meta-analysis compared perioperative and functional outcomes between double-barrel uro-colostomy (DBUC) and ileal conduit (IC) urinary diversion techniques across four studies encompassing 164 patients [18,19,24,25]. Despite longstanding concerns about combining urinary and faecal diversion into a single stoma, our pooled analysis found no statistically significant differences in major complications (Clavien–Dindo grade III–IV), suggesting that DBUC may be a safe and effective alternative to IC, offering comparable or even favourable perioperative outcomes in selected patients. However, given the small number of studies and inherent methodological limitations, these results must be interpreted with caution.

From a technical perspective, the avoidance of small bowel anastomosis in DBUC represents a potentially significant surgical advantage, particularly in the context of a previously irradiated pelvis, which is common among patients undergoing pelvic exenteration [26,27]. Pelvic irradiation is known to impair tissue vascularity and healing capacity, increasing the risk of anastomotic complications [28]. Across the included studies, a substantial proportion of patients had received pre-operative radiotherapy, with reported rates ranging from 60% to 100%. Importantly, a higher proportion of patients in the DBUC groups were irradiated compared to those in the IC cohorts (77–100% vs. 60–100%) [18,19,24,25]. By utilising healthy, well-vascularised, and typically non-irradiated segments of proximal colon for urinary reconstruction, DBUC may mitigate some of the anastomotic complications more commonly seen when irradiated small bowel is employed, as in ileal conduits [29]. This theoretical benefit is supported by our pooled analysis, which demonstrated a significantly higher risk of urinary leak in the IC group (RR 2.52, 95% CI 1.02–6.20, *p* = 0.04). Clinically, these findings suggest that patient selection should be individualized. DBUC may be particularly advantageous in patients with extensive pelvic irradiation, limited small bowel availability, or when preserving abdominal wall musculature is important. Conversely, ileal conduit diversion remains suitable for cases where the ileum is healthy and patient preference favours separation of urinary and faecal stomas. Integrating these considerations into preoperative planning may optimize reconstruction choice and postoperative outcomes.

In addition to improving tissue integrity and healing, avoiding an ileal anastomosis also simplifies the operative field [20,30]. This reduction in surgical complexity may translate to shorter operative time. Backes et al. reported longer operative times in the IC group (720 min) compared to the DBUC group (610 min). Chokshi et al. found comparable operative durations between the two groups (approximately 547–549 min). Nguyen et al. paradoxically reported a mean operative time of 566 min for DBUC compared to 415 min for IC group, but with considerably more complex patients and more flaps in the DBUC group. These differences likely reflect non-standardized protocols, institutional preferences, and surgeon experience. Such factors introduce performance bias and limit direct comparison. Nevertheless, by reducing operative complexity and mitigating risks in compromised tissues, DBUC has a compelling technical rationale, particularly after pelvic irradiation.

The single-stoma approach of DBUC emerges as its most compelling benefit, both from a practical and patient-centred perspective [31]. Managing two separate stomas can pose significant challenges for patients, particularly in terms of appliance burden, skin care, and daily routines [32]. Qualitative data from small series and narrative reviews highlight patient preference for one stoma over two [23,33]. Similarly, a literature review of 300 DBUC patients emphasised that the one-stoma approach is not only easier for patients to manage, but also simplifies the training and support required from stoma care teams [8]. In patients with compromised dexterity, poor vision, or limited caregiver support, this reduction in appliance complexity may be particularly advantageous. These findings are consistent with the clinical impression that DBUC reduces ostomy-related complications such as skin irritation, leakage, and poor appliance fit due to conflicting stoma positions. Future studies are needed to evaluate differences in patient satisfaction and quality of life between individuals living with a single stoma versus those with dual stomas.

Renal safety profile is another important consideration when comparing urinary diversion techniques. Pyelonephritis and electrolyte disturbances are well-recognised complications following urinary reconstruction, particularly in the context of bowel–urinary tract anastomoses [14,15]. In our pooled analysis, pyelonephritis occurred in 15.28% of DBUC patients compared to 42.00% in the IC group, although this difference did not reach statistical significance (RR 1.37, 95% CI: 0.81–2.30, *p* = 0.24). The lower absolute rate in the DBUC group may relate to differences in bowel colonisation patterns or conduit configuration, but the small sample sizes limit definitive interpretation. Electrolyte derangements often reflect chronic metabolic acidosis from urinary contact with bowel mucosa and were also comparable between groups (RR 1.21, 95% CI 0.54–2.67, *p* = 0.64) [33,34]. These findings suggest that, at least in the short- to medium-term follow-up available, DBUC does not appear to confer an excess renal or metabolic risk compared to IC. However, longer-term surveillance studies are warranted to assess whether differences emerge over time, particularly in renal function decline and metabolic complications.

However, certain theoretical drawbacks to DBUC remain. A major concern is the potential long-term risk of malignancy at the uretero-colonic junction [24,25,26,27,28]. Historical studies have reported a higher incidence of adenomatous polyps and carcinomas at this site in patients with ureterosigmoidostomies [24,25,26]. For example, one study with long-term follow-up of 75 patients who underwent ureterosigmoidostomy revealed an 8.5–10.5-fold increased risk of colonic carcinoma relative to the general population, and direct biopsy confirmed malignancy at the uretero-colonic junction [27]. Subsequent literature reports incidence rates up to 100- to 500-fold greater than baseline, with latency often exceeding 20 years [28]. These findings raised concerns about the carcinogenic potential of prolonged exposure of colonic mucosa to urinary constituents. Although these risks were primarily observed in older, refluxing ureterosigmoidostomies, they remain a theoretical concern in contemporary colonic urinary diversions, including double-barrel uro-colostomy. Importantly, it must be noted that malignancy is not limited to colonic urinary diversions. Late secondary malignancies have also been reported in ileal urinary conduits [34,35]. Studies have identified cases of small-bowel carcinoma and transitional cell carcinoma arising at the ileal segment of urinary diversions, highlighting that the ileum is not immune to long-term malignant transformation developing in patients with ileal conduits and neobladders, with latency periods extending up to 45 years [35,36]. These findings emphasise that no segment of bowel used in urinary reconstruction is entirely without oncological risk, and all conduit types warrant long-term surveillance. While none of the studies included in our review reported cases of malignant transformation, follow-up durations were limited. Despite the absence of established long-term surveillance programmes for DBUC and IC urinary diversions, continued follow-up remains important to identify rare but clinically significant late complications.

Patient reported quality of life is another long standing debated drawback to DBUC. Although the urinary and faecal streams are anatomically separated, the shared stoma may produce effluent that is less predictable in volume or consistency, particularly if patients experience altered bowel function. This may affect pouching, skin care, and patient comfort. Comparative quality of life data between DBUC and IC remain sparse. However, a number of studies investigating quality of life in patients post-DBUC formation report favourable outcomes [37,38]. Kesteren et al. included a series of 20 patients who were followed post-DBUC formation [37]. In terms of urinary and faecal diversion, the main issue that arose was related to high urinary frequency. Mean stoma quality of life score in this cohort was 56.1 (scale of 0 to 100: 0 indicating poor QoL, 100 indicating good QoL). A one-year follow-up decision regret scale was also completed in this cohort with no patient expressing regret. Queiroz et al. published another small series of QoL data on 9 patients [38]. Similarly to Kesteren et al., the most common quality of life complaint was the need for frequent stoma bag changes, especially overnight. However, all patients assessed reported ease of care of the stoma and personal hygiene. While data from such studies are promising in terms of reflecting positive quality of life outcomes, these findings must be interpreted cautiously given the small cohort sizes and lack of comparative analysis with IC.

There are significant limitations to this study. All included studies were retrospective, with small sample sizes and limited follow-up. Surgical technique and case selection likely varied between centres and were often poorly described. Crucially, it is unclear whether the same surgeons performed both DBUC and IC in each centre. Given that DBUC may have been adopted later, with increasing institutional experience or specific surgeon preference, this introduces a risk of confounding by expertise. For instance, if one group performs predominantly DBUC and another IC, differences in outcomes may reflect surgical familiarity rather than true differences between the techniques. This issue is compounded by the small number of studies (n = 4), which precludes robust subgroup analysis or assessment of learning curves, and therefore further prospective studies are needed to provide more robust data. In addition, given the small number of studies identified, publication bias cannot be excluded. Although a funnel plot was generated to explore potential publication bias (Supplementary Figures S3–S5), the limited number of studies (<10) reduces the reliability of visual or statistical interpretation. It is possible that positive findings were more likely to be published, and future multicenter prospective studies are required to validate these results in broader cohorts.

In conclusion, while limitations in current evidence preclude definitive recommendations, the findings from this systematic review suggest that DBUC may be a viable urinary diversion strategy following pelvic exenteration. It may offer distinct advantages in surgical logistics, postoperative recovery, and patient care, particularly in individuals with prior pelvic radiotherapy or where preservation of the abdominal wall is critical. However, patient numbers are small. Thus, definitive conclusions cannot be made and further prospective, comparative studies incorporating validated patient-reported outcome measures are needed to establish long-term safety and functional equivalence or superiority to ileal conduit diversion. Nonetheless, in appropriately selected surgical candidates, DBUC may be considered a viable option for urinary reconstruction following pelvic exenteration.

## 5. Conclusions

This systematic review and meta-analysis demonstrates that double-barrel uro-colostomy (DBUC) is a feasible and safe alternative to the ileal conduit (IC) for urinary diversion following pelvic exenteration. Across four comparative studies, DBUC was associated with a significantly lower rate of urinary leaks and similar incidences of pyelonephritis, electrolyte disturbance, and major postoperative complications. The technical advantages of avoiding a small bowel anastomosis and consolidating diversion into a single stoma may offer both operative and patient-centred benefits, particularly in individuals with prior pelvic irradiation or complex reconstructions.

While these findings are encouraging, current evidence remains limited by small sample sizes, retrospective design, and short follow-up periods. Future prospective, multicentre studies incorporating long-term oncological, renal, and quality-of-life outcomes are warranted. Until such data are available, DBUC should be considered a viable reconstructive option in carefully selected patients undergoing pelvic exenteration, guided by surgical expertise and individual patient factors.

## Figures and Tables

**Table 1:** Summary of Baseline and Operative Variables.

Study	Group	*n*	Age (Mean ± SD or Median [Range])	BMI (Mean or Median)	Pre-op RT (%)	Type of Exenteration (%)	Operative Time (min)	Length of Stay (Days) Median ([Range])	Flap Use (% and Type)
Lago et al. [24]	DBUC	20	62 ± 11	26 ± 6.5 (not group-specific)	84 (not group-specific)	Total 65, Anterior 20, Posterior 15 (not group-specific)	412 ± 124 (not group-specific)	16 (3–38)	e-VRAM 30%, Omental 44%, Gracilis 3%, V-Y 7% (not group-specific)
	IC	29	57 ± 11	-	-	-	-	22 (7–72)	-
Chokshi et al. [25]	DBUC	43	56 [38–79]	-	77	Total 100	547 [240–864]	14 [6–57]	-
	IC	10	62 [46–80]	-	60	Total 100	549 [279–720]	16.5 [8–37]	-
Backes et al. [20]	DBUC	12	54 [42–72]	29	100	-	610 [406–853]	14.5 [10–57]	-
	IC	11	57 [28–76]	25	100	-	720 [635–1003]	26 [14–37]	-
Nguyen et al. [19]	DBUC	16	56.6 ± 18.1	26.5 [17–36]	93.8	Total 100	566.3 ± 204.6	18.5 [8–73]	None 6.3%, VRAM + other 37.5%, Omental 31.3%, VRAM 25%,
	IC	23	66.5 ± 11.5	26 [22–45]	65.2	Total 52.2, Modified Total 8.7, Anterior 30.4, Other 8.7	414.6 ± 167.9	16 [9–57]	None 54.5%, VRAM 18.2%, Omental 13.6%, VRAM + other 4.5%, Gracilis 9.1%

**Table 2:** Summary of Perioperative Outcomes Comparing DBUC and IC.

Study	Group	n	Urinary Leaks	Pyelonephritis	Electrolyte Disturbances	Urinary Fistula	Enteric Fistula
Lago et al. (2023) [24]	DBUC	17	0	9 (53%)	3 (18%)	2 (12%)	2 (12%)
	IC	29	1 (3.4%)	17 (59%)	7 (24%)	0 (0%)	8 (28%)
Chokshi et al. (2011) [25]	DBUC	43	8 (18.6%)	1 (2.3%)	-	6 (14%)	13 (30.2%)
	IC	10	3 (30%)	1 (10%)	-	6 (60%)	1 (10%)
Backes et al. (2012) [20]	DBUC	12	0	1 (8%)	0 (0%)	-	-
	IC	11	5 (45%)	3 (27%)	1 (9%)	-	-
Nguyen et al. (2023) [19]	DBUC	16	1 (6.25%)	-	4 (25.00%)	-	0 (0%)
	IC	23	2 (8.70%)	-	5 (21.74%)	-	1 (4.35%)

## Supplementary Materials

The following supporting information can be downloaded at: https://www.mdpi.com/article/10.3390/cancers17213479/s1, Figure S1: Risk of bias by Category; Figure S2: Risk of bias by Study; Figure S3: Funnel plot of comparison: Urinary Leak; Figure S4: Funnel plot of comparison: Pyelonephritis; Figure S5: Funnel plot of comparison: Electrolyte Derangement; Table S1: Summary of studies Included; Table S2: Summary of Clavien–Dindo Grade III–IV Postoperative Complications.

## Abbreviations

DBUC	Double-Barrel Uro-Colostomy
IC	Ileal Conduit
PRISMA	Preferred Reporting Items for Systematic Reviews and Meta-Analyses
RR	Risk Ratio
CI	Confidence Interval
LOS	Length of Stay
QoL	Quality of Life
TPE	Total Pelvic Exenteration
VRAM	Vertical Rectus Abdominis Myocutaneous (flap)
