# Supplementary material for: Double-Barrel Uro-Colostomy Versus Ileal Conduit for Urinary Diversion After Pelvic Exenteration: A Systematic Review and Meta-Analysis of Comparative Outcomes
Source: Cancers (Basel). 2025 Oct 29;17(21):3479. doi: 10.3390/cancers17213479 (PMC12607525; doi:10.3390/cancers17213479)
Supplement: Supplementary file 1 [file cancers-17-03479-s001.zip › cancers-3908150-supplementary.pdf]

cancers

Supplementary File

Figures

Figure S1: Risk of bias by Category

Figure S2: Risk of bias by Study

Figure S3: Funnel plot of comparison: Urinary Leak

Figure S4: Funnel plot of comparison: Pyelonephritis

Figure S5: Funnel plot of comparison: Electrolyte Derangement

Tables

Table S1: Summary of studies Included

Table S2: Summary of Clavien-Dindo Grade III–IV Postoperative Complications

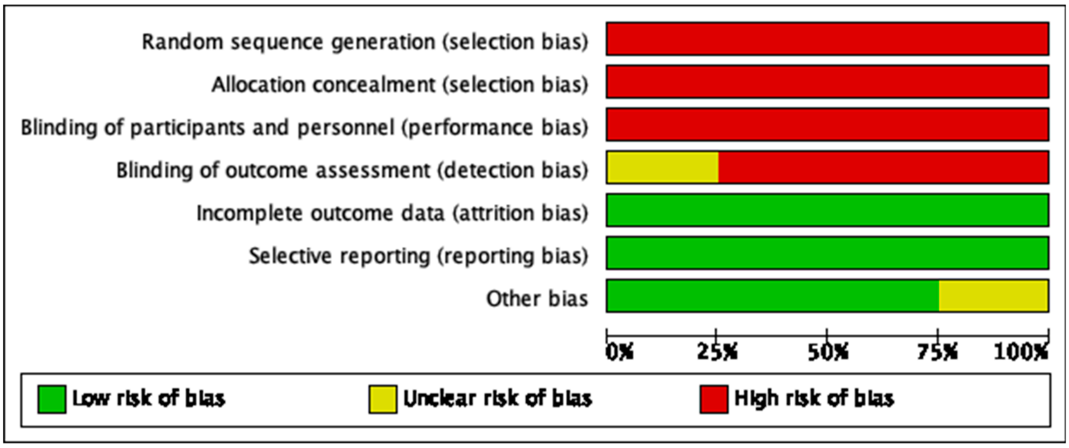

Figure S1. Risk of bias by Category.

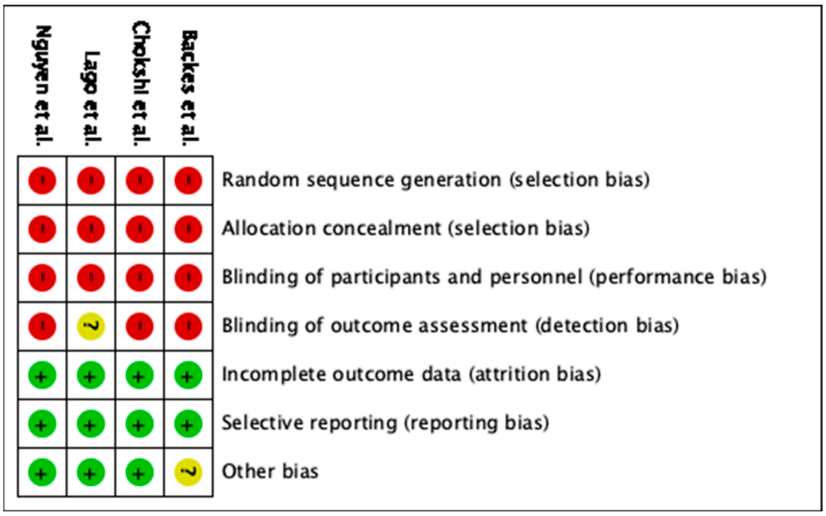

Figure S2. Risk of bias by Study.

Table S1. Summary of studies Included.

Reference	year	Country	Journal	Study type	Sample size	Comparison	Tumour Origin
Lago et al.[1]	2023	Spain	European Journal of Obstetrics & Gynecology and Reproductive Biology	Retrospective cohort	49	IC=29<br>DBUC=20	Gynae=49
Chokshi et al. [2]	2011	USA	Urology	Retrospective cohort	53	IC=10<br>DBUC=43	Colorectal=36<br>Gynae=6<br>Urological=5<br>Leimyosarcoma=3<br>Anal SCC=2<br>Suspected Malignancy=1
Backes et. al. [3]	2012	USA	Gynecological Oncology	Retrospective cohort	23	IC=11<br>DBUC=12	Gynaecological=23
Nguyen et al. [4]	2023	Australia	ANZ Journal of Surgery	Retrospective cohort	39	IC=23<br>DBUC=16	Colorectal=24<br>Gynae =10<br>Urology=1<br>Benign=1

Table S2. Summary of Clavien-Dindo Grade III–IV Postoperative Complications in DBWC vs IC Following Pelvic Exenteration.

Study	Group	n	CD Grade III	CD Grade IV
**Lago et al. (2023)** [1]	DBUC	17	8 (47%)	3 (18%)
	IC	29	16 (55%)	13 (45%)
**Backes et al. (2012)** [3]	DBUC	12	-	-
	IC	11	-	-
**Nguyen et al. (2023)** [4]	DBUC	16	3 (18.75%)	0 (0%)
	IC	23	2 (8.70%)	1 (4.35%)
**Chokshi et al. (2011)** [2]	DBUC	43	21 (48.84%)	10 (23.26%)
	IC	10	5 (50%)	1 (10%)

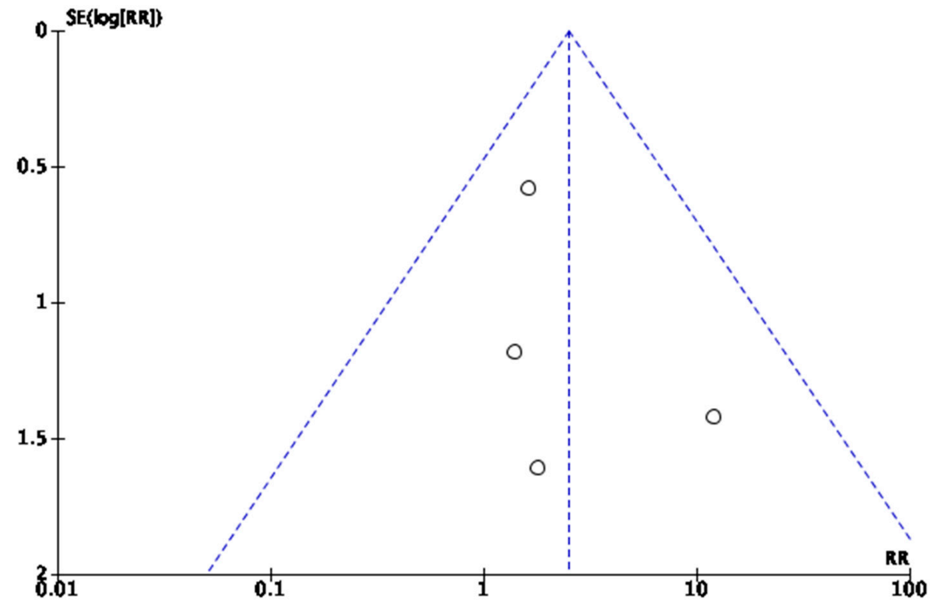

Figure S3. Funnel plot of comparison: Urinary Leak.

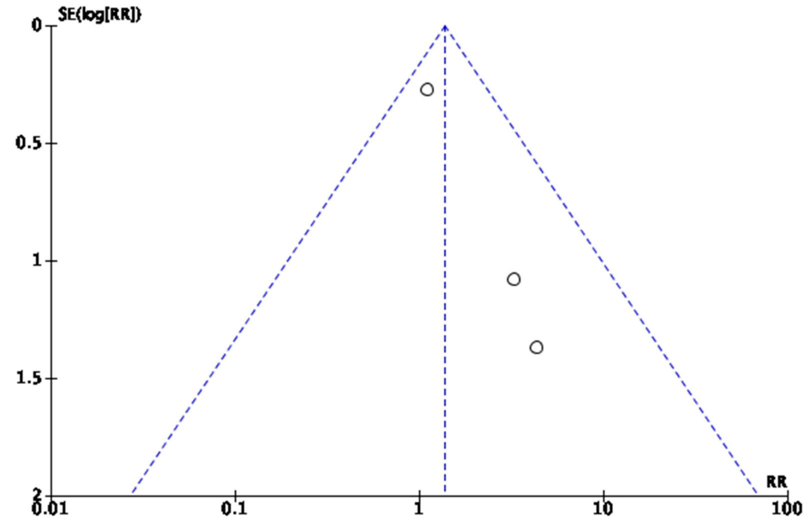

Figure S4. Funnel plot of comparison: Pyelonephritis.

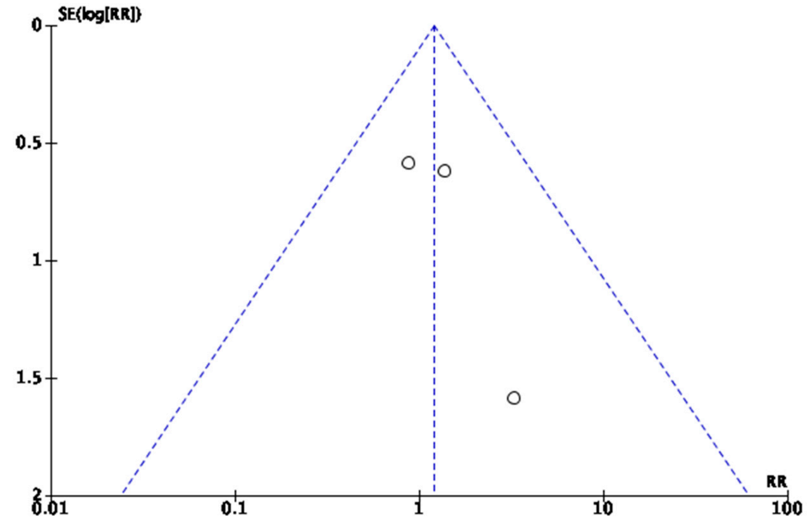

Figure S5. Funnel plot of comparison: Electrolyte Derangement.

References

1. Lago, V.; Pradillo Aramendi, T.; Segarra-Vidal, B.; Padilla-Iserte, P.; Matute, L.; Gurrea, M.; Pontones, J.L.; Delgado, F.; Domingo, S. Comparation between the Bricker ileal conduit vs double-barrelled wet colostomy after pelvic exenteration for gynaecological malignancies. *Eur. J. Obstet. Gynecol. Reprod. Biol.* **2023**, *282*, 140–145.
2. Chokshi, R.J.; Kuhrt, M.P.; Arrese, D.; Parks, L.; Johnson, M.; Martin, E.W. Single-institution experience comparing double-barreled wet colostomy to ileal conduit for urinary and fecal diversion. *J. Clin. Oncol.* **2011**, *29*, 542–542.
3. Backes, F.J.; Tierney, B.J.; Eisenhauer, E.L.; Bahnson, R.R.; Cohn, D.E.; Fowler, J.M. Complications after double-barreled wet colostomy compared to separate urinary and fecal diversion during pelvic exenteration: Time to change back? *Gynecol. Oncol.* **2013**, *128*, 60–64.
4. Nguyen, T.M.; Traeger, L.; Vather, R.; Overall, B.; Cho, J.; Sammour, T. Double barrelled uro-colostomy versus Ileal conduit for urinary diversion following pelvic exenteration: A single centre experience. *ANZ J. Surg.* **2023**, *93*, 2450–2456.
